# Comparative genomic analysis reveals significant enrichment of mobile genetic elements and genes encoding surface structure-proteins in hospital-associated clonal complex 2 *Enterococcus faecalis*

**DOI:** 10.1186/1471-2180-11-3

**Published:** 2011-01-04

**Authors:** Margrete Solheim, Mari C Brekke, Lars G Snipen, Rob JL Willems, Ingolf F Nes, Dag A Brede

**Affiliations:** 1 Laboratory of Microbial Gene Technology and Food Microbiology, Department of Chemistry, Biotechnology and Food Science, The Norwegian University of Life Sciences, N-1432 Ås, Norway; 2 Section for Biostatistics, Department of Chemistry, Biotechnology and Food Science, The Norwegian University of Life Sciences, N-1432 Ås, Norway; 3 Department of Medical Microbiology, University Medical Center Utrecht, Utrecht, The Netherlands

## Abstract

**Background:**

Enterococci rank among the leading causes of nosocomial infections. The failure to identify pathogen-specific genes in *Enterococcus faecalis *has led to a hypothesis where the virulence of different strains may be linked to strain-specific genes, and where the combined endeavor of the different gene-sets result in the ability to cause infection. Population structure studies by multilocus sequence typing have defined distinct clonal complexes (CC) of *E. faecalis *enriched in hospitalized patients (CC2, CC9, CC28 and CC40).

**Results:**

In the present study, we have used a comparative genomic approach to investigate gene content in 63 *E. faecalis *strains, with a special focus on CC2. Statistical analysis using Fisher's exact test revealed 252 significantly enriched genes among CC2-strains. The majority of these genes were located within the previously defined mobile elements *phage03 *(n = 51), *efaB5 *(n = 34) and a *vanB *associated genomic island (n = 55). Moreover, a CC2-enriched genomic islet (EF3217 to -27), encoding a putative phage related element within the V583 genome, was identified. From the draft genomes of CC2-strains HH22 and TX0104, we also identified a CC2-enriched non-V583 locus associated with the *E. faecalis *pathogenicity island (PAI). Interestingly, surface related structures (including MSCRAMMs, internalin-like and WxL protein-coding genes) implicated in virulence were significantly overrepresented (9.1%; *p *= 0.036, Fisher's exact test) among the CC2-enriched genes.

**Conclusion:**

In conclusion, we have identified a set of genes with potential roles in adaptation or persistence in the hospital environment, and that might contribute to the ability of CC2 *E. faecalis *isolates to cause disease.

## Background

For many years, *Enterococcus faecalis *was considered as an intestinal commensal, which only sporadically caused opportunistic infections in immunocompromised patients. During the last thirty years, however, *E. faecalis *has gained notoriety as one of the primary causative agents of nosocomial infections [1,2], including urinary tract infections, endocarditis, intra-abdominal infections and bacteremia. The ability of *E. faecalis *to cause infection has been connected to inherent enterococcal traits, enabling the bacterium to tolerate diverse and harsh growth conditions. Moreover, several putative enterococcal virulence factors have been characterized (reviewed in [3]), and the role of these virulence factors in pathogenicity have been further established in various animal infection models [4-8] and cultured cell lines [9,10]. Reportedly, several of the proposed virulence determinants are enriched among infection-derived *E. faecalis *and/or *E. faecium *isolates, including *esp *(enterococcal surface protein) [11], *hyl *(hyaluronidase) [12], genes encoding collagen binding adhesins [13,14] and other matrix-binding proteins [15], and pilin loci [16,17]. On the other hand, recent studies on enterococcal pathogenicity have shown that a number of the putative virulence traits are present not only in infectious isolates but also in animal and environmental isolates [18-23]. This widespread distribution of putative virulence determinants in enterococcal isolates strongly suggest that enterococcal pathogenicity is not a result of any single virulence factor, but rather a more intricate process. Indeed, the virulence potential of the newly sequenced laboratory strain *E. faecalis *OG1RF was, despite its lack of several factors, comparable to that of the clinical isolate *E. faecalis *V583 [24]. Bourgogne et al. [24] proposed a scenario where the virulence of V583 and OG1RF may be linked to genes that are unique to each of the two strains, but where the combined endeavor of the different gene-sets result in the ability to cause infection.

Population structure studies of *E. faecalis *by multilocus sequence typing (MLST) have previously defined distinct clonal complexes (CC) of *E. faecalis *enriched in hospitalized patients (CC2, CC9, CC28 and CC40), designated high-risk enterococcal clonal complexes (HiRECCs) [25,26]. In one of our previous studies, we reported an overall correlation between MLST and Bayesian phylogenetic analysis of gene content as revealed by microarray-based comparative genomic hybridization (CGH) [27]. This observation led us to speculate whether the virulence of different HiRECCs may be due to lineage-specific gene sets. In the present study we have used the comparative genomics approach to further investigate variation in gene content within *E. faecalis*, with a special focus on CC2. This complex was chosen on the basis of previous Bayesian-based phylogenetic reconstruction [27]. CC2 is equivalent to the previously designated BVE complex, and comprises several clinically important *E. faecalis *isolates, including the first known beta-lactamase producing isolate HH22, the first U.S. vancomycin-resistant isolate V583, and pathogenicity island (PAI)-harboring clinical bacteremia isolate MMH594 [26,28,29]. This CC represents a globally dispersed hospital-associated lineage, and identification of CC2-enriched genes may unravel novel fitness factors implicated in survival and spread of *E. faecalis *clones in the hospital environment.

## Results and discussion

### Overall genomic diversity

To explore the genetic diversity among *E. faecalis*, BLAST comparison was performed with 24 publicly available sequenced draft genomes, including the two CC2-strains TX0104 (ST2), which is an endocarditis isolate, and HH22 (ST6; mentioned above) against the genome of strain V583, which is also a ST6 isolate. The number of V583 genes predicted to be present varied between 2385 (OG1RF) and 2831 (HH22) for the 24 strains (Additional file 1). In addition, we used CGH to investigate variation in gene content within 15 *E. faecalis *isolated in European hospital environments, with a special focus on a hospital-adapted subpopulation identified by MLST (CC2). Of the 3219 V583 genes represented on the array, the number of V583 orthologous genes classified as present ranged from 2359 (597/96) to 2883 (E4250). Analysis of the compiled data set (*in silico *and CGH), revealed a total of 1667 genes present in all strains, thus representing the *E. faecalis *core genome. None of the annotated V583 genes were found to be divergent in all the isolates analyzed.

### Putative CC2-enriched elements

**Figure 1:**
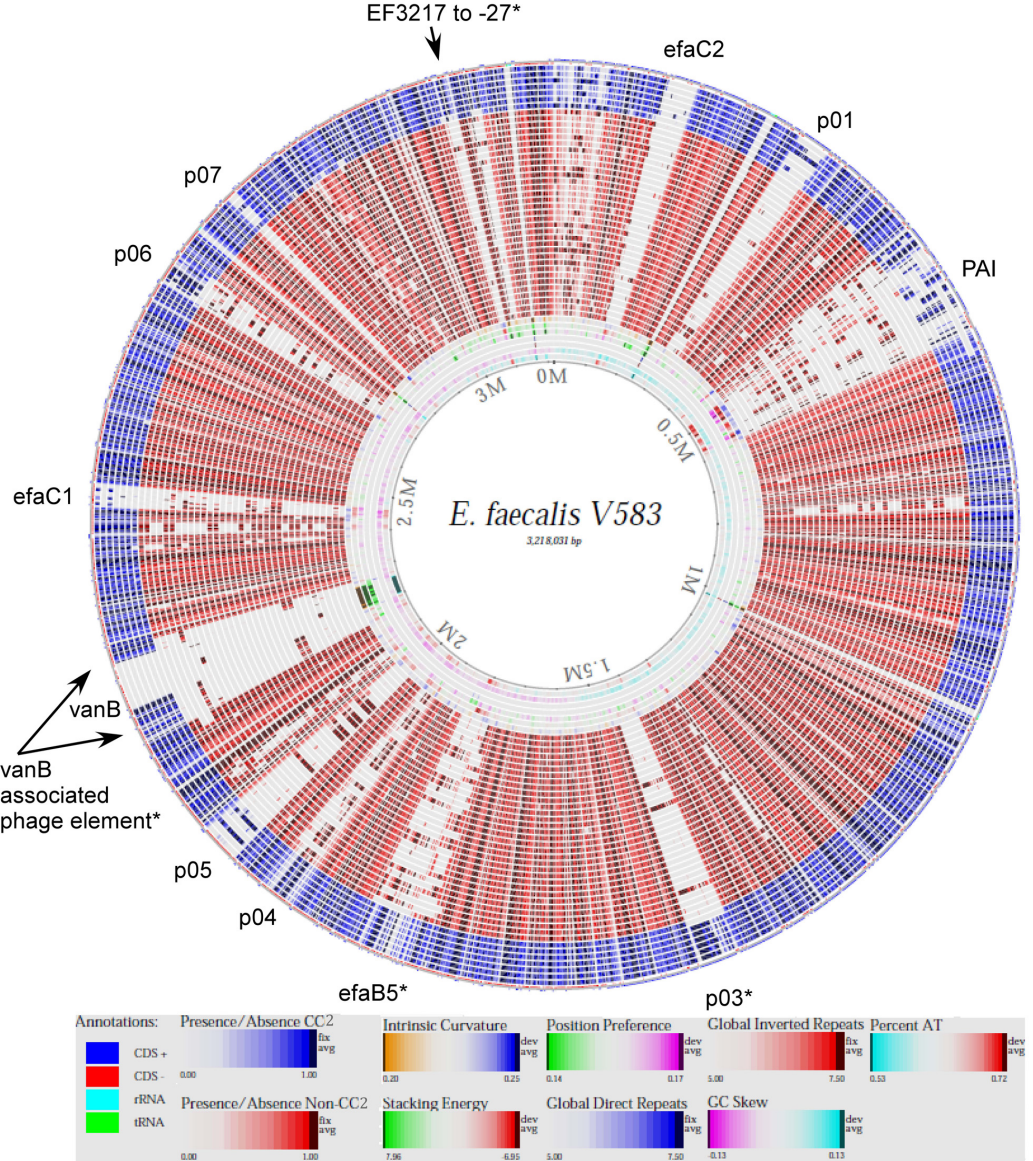
**Genome-atlas presentation of CGH data compared to the V583 genome and arranged by clonal relationship according to MLST**. From inner to outer lanes: 1) percent AT, 2) GC skew, 3) global inverted repeats, 4) global direct repeats, 5) position preference, 6) stacking energy, 7) intrinsic curvature, 8) 189, 9) LMGT3208, 10) LMGT3407, 11) 92A, 12) 29C, 13) E1960, 14) 111A, 15) 105, 16) E2370, 17) 84, 18) 383/04, 19) E1188, 20) Vet179, 21) EF1841, 22) E1807, 23) LMGT3143, 24) LMGT3405, 25) OG1RF, 26) 2426/03, 27) LMGT3406, 28) 85, 29) E1052, 30) 1645, 31) LMGT3209, 32) LMGT2333, 33) 597/96, 34) 62, 35) Vet138, 36) 266, 37) UC11/96, 38) Symbioflor 1, 39) 3339/04, 40) 82, 41) E1834, 42) E4250, 43) LMGT3303, 44) 158B, 45) MMH594, 46) 372-56, 47) 609/96 and 48) annotations in V583. Elements enriched in CC2-strains are indicated with an asterisk.

In a previous study, we identified a set of potential pathogen-specific genes, which were entirely divergent in a collection of commensal baby isolates [27]. None of these genes were found to be present in all hospital-related isolates analyzed in the present study, neither was any gene found to be unique to any HiRECC. In order to identify genes specifically enriched among strains belonging to CC2, data from the present study were supplemented with hybridization data from an additional 24 strains of various origins ([27,30] and M. Solheim, unpublished data). The additional data sets were obtained by hybridization to the same array as described above. All together, data from a total of 63 strains were analyzed, in addition to V583 (Table 1). A genome-atlas presentation of the gene content in all the strains analyzed by CGH compared to the V583 genome is shown in Figure 1.

**Table 1:** Enterococcus faecalis isolates used in this study. CC; clonal complex, CGH; comparative genomic hybridization, MLST; multilocus sequence typing, S; singleton, ST; sequence type.

Strain	Year	Country	Source	MLST		Application	Reference
				ST	CC		
TX0104		USA	Clinical	2	2	*In silico*	[65]
609/96	1996	Poland	Wound	6	2	CGH, PCR	[25]
372-56	2007	Norway	Blood	6	2	CGH, PCR	
226B	2005	Norway	Feces	6	2	PCR	[27]
368-42	2007	Norway	Blood	6	2	PCR	
442/05	2005	Poland	CSF	6	2	PCR	[25]
E1828	2001	Spain	Blood	6	2	PCR	[26]
MMH594	1985	USA	Clinical	6	2	CGH^C^, PCR	[66]
V583	1989	USA	Blood	6	2	CGH, PCR	[67]
158B	2005	Norway	Feces	6	2	CGH^B^, PCR	[27]
HH22	≤1982	USA	Urine	6	2	*In silico*	[29]
LMGT3303				6	2	CGH^D^, PCR	
E1834	2001	Spain	Blood	51	2	CGH, PCR	[26]
E4250	2007	Netherlands	Feces	183	2	CGH, PCR	
HIP11704	2002	USA	Clinical	4	4	*In silico*	[68]
E1841	2001	Spain	Blood	9	9	CGH, PCR	[26]
Vet179	1999	Norway	Dog_urine	9	9	CGH^D^, PCR	[69]
CH188	1980s	USA	Liver	9	9	*In silico*	[70]
E1807	2002	Spain	Feces	17	9	CGH, PCR	[26]
X98	1934		Feces	19	19	*In silico*	[71]
OG1RF	≤1975	USA	Oral	1	21	CGH^C^, PCR	[72]
E1960	2001	Spain	Feces	8	21	CGH, PCR	[26]
T8	≤1992	Japan	Urine	8	21	*In silico*	[73]
2426/03	2003	Poland	Feces	21	21	CGH, PCR	[25]
ATCC 29200	≤1974	Canada	Urogenital	21	21	*In silico*	[74]
T1	≤1950			21	21	*In silico*	[73]
LMGT3406	1999	Denmark	Poultry_feces	22	21	CGH^D^, PCR	
111A	2005	Norway	Feces	161	21	CGH^B^, PCR	[27]
TX1322		USA		161	21	*In silico*	[65]
3339/04	2004	Poland	Blood	23	25	CGH, PCR	[25]
UC11/46		Finland	Feces	97	25	CGH, PCR	[19]
189	2002-2003	Norway	Feces	162	25	CGH^B^, PCR	[27]
Symbioflor 1		Germany	Feces	248	25	CGH^C^, PCR	[75]
T2	≤1992	Japan	Urine	11	28	*In silico*	[73]
E1188	1997	Greece	Blood	28	28	CGH, PCR	[26]
383/04	2004	Poland	Blood	87	28	CGH, PCR	[25]
E1052		Netherlands	Feces	30	30	CGH^D^, PCR	
85	2008	Norway	Feces	30	30	CGH^B^, PCR	[27]
597/96	1996	Poland	Ulcer	40	40	CGH, PCR	[25]
LMGT2333		Iceland	Fish	40	40	CGH^D^, PCR	
JH1	≤1974	United Kingdom	Clinical	40	40	*In silico*	[76]
LMGT3209		Greece	Food_cheese	40	40	CGH^D^, PCR	
1645	2007	Denmark	Blood	220	40	CGH, PCR	
29C	2004	Norway	Feces	44	44	CGH^B^, PCR	[27]
92A	2005	Norway	Feces	44	44	CGH^B^	[27]
DS5	≤1974			55	55	*In silico*	[77]
E2370		Hungary	Wound	16	58	CGH, PCR	
105	2002-2003	Norway	Feces	16	58	CGH^B^, PCR	[27]
D6		Denmark	Pig	16	58	*In silico*	[31]
E1Sol	1960s	Solomon Islands	Feces	93	93	*In silico*	[78]
Merz96	2002	USA	Blood	103	103	*In silico*	[79]
R712		USA	Clinical	103	103	*In silico*	[65]
S613		USA	Clinical	103	103	*In silico*	[65]
LMGT3405	1999	Denmark	Poultry_feces	116	116	CGH^D^, PCR	
LMGT3407	1999	Denmark	Poultry_feces	34	121	CGH^D^, PCR	
Fly1	2005	USA	Drosophila	101	101^A^	*In silico*	[31]
Vet138	1998	Norway	Dog_ear	164	119^A^	CGH^D^, PCR	[69]
82	2008	Norway	Poultry_feces	65	S	CGH^D^, PCR	
T11	≤1992	Japan	Urine	65	S	*In silico*	[73]
62	2002-2003	Norway	Feces	66	S	CGH^B^, PCR	[27]
ATCC 4200	1926		Blood	105	S	*In silico*	
AR01/DG	2001	New Zealand	Dog	108	S	*In silico*	[80]
266	2002-2003	Norway	Feces	163	S	CGH^B^, PCR	[27]
LMGT3143		Spain	Animal_wood pigeon	165	S	CGH^D^, PCR	
LMGT3208		Greece	Food_cheese	166	S	CGH^D^, PCR	
84	2008	Norway	Poultry_feces	249	S	CGH^D^, PCR	
TuSoD ef11		USA	Clinical	364	S	*In silico*	[65]

^A^Clonal complexes were no predicted founder was proposed by eBURST.

^B^In Solheim et al. 2009.

^C^In Vebø et al. 2010.

^D^MS, unpublished work.

By Fisher's exact testing (*q *< 0.01), 252 genes were found to be more prevalent among CC2-strains than in non-CC2-strains (Additional file 2). The CC2-enriched genes included large parts of *phage03 *(*p03*; n = 51), *efaB5 *(n = 34) and a phage-related region identified by McBride et al. [31](EF2240-82/EF2335-51; n = 55), supporting the notion that the *p03 *genetic element may confer increased fitness in the hospital environment [27]. Indeed, prophage-related genes constituted a predominant proportion of the CC2-enriched genes (55.5%; *p *< 2.2e-16, Fisher's exact test). Interestingly, the Tn*916*-like *efaB5 *element has previously also been suggested to play a role in niche adaptation (Leavis, Willems et al. unpublished data): CGH analysis identified an *efaB5*-orthologous element in *E. faecium *that appeared to be common for HiRECC *E. faecalis *and CC17 *E. faecium*, a hospital-adapted subpopulation identified by MLST. To further confirm the presence of the relevant MGEs in *E. faecalis*, we used PCR combining internal primers with primers targeting the genes flanking *p03*, *efaB5 *and the *vanB*-associated phage-related element in V583, to monitor conserved V583 junctions on either side of the elements in 44 strains (Table 1). Seven strains contained the junctions on both sides of *p03*, of which six strains were CC2-strains. Eleven strains were positive for the junctions on both sides of *efaB5*, including nine CC2-strains, while thirteen strains gave positive PCR for both junctions of the phage-related element surrounding *vanB*, of which eleven strains belonged to CC2 (Additional file 3). These results substantiate the theory of *p03*, *efaB5 *and the *vanB*-associated phage as CC2-enriched elements.

A total of 178 of the 252 putative CC2-enriched genes identified here, were associated with previously defined MGEs identified in V583 [32]. In addition to *p03*, *efaB5 *and the *vanB*-surrounding phage element, these included *p01 *(n = 5), PAI (n = 7), *p04 *(n = 21), *p06 *(n = 1) and pTEF1 and pTEF2 (n = 5) (Additional file 2). In addition, a ten-gene cluster (EF3217 to -27) with significant GC skew compared to the genome-average (31.6 and 37.4%, respectively), was found to be significantly more frequent in strains belonging to CC2 than in non-CC2 strains. The deviation in GC content suggests that this genetic element may also be of foreign origin. This notion was further supported by the sequence similarities of several of the genes with known phage-related transcriptional regulators (EF3221, EF3223 and EF3227). Moreover, EF3221 to -22 showed high degree of identity (>85%) to EfmE980_2492 to -93 of the newly sequenced *Enterococcus faecium *E980 [33]. EfmE980_2492 holds a domain characteristic of the aspartate aminotransferase superfamily of pyridoxal phosphate-dependent enzymes. Interestingly, EF3217 encodes a putative helicase, while EF3218 encodes a putative MutT protein, both with implications in DNA repair [34,35]. A potential role of these genes in protection against oxidative DNA damage induced in the hospital environment and during infection is plausible. To further investigate the distribution of EF3217 to -27 in *E. faecalis*, 44 strains were screened by PCR (Additional file 3): 10 CC2-strains held all ten genes, while 19 strains including two CC2-strains were devoid of the entire element. Moreover, 2 strains contained EF3225 only, 3 strains contained EF3217 to -18, while 8 strains, including OG1RF, contained EF3226 only. The two latter patterns of presence and divergence of EF3217 to -27 were also obtained with BLASTN analysis of TX0104 and OG1RF, respectively, corroborating that these are indeed genuine polymorphisms in this locus. Notably, in the OG1RF genome five more genes (OG1RF_0214 to -18) are also located between the homologs of EF3216 and EF3230 [24], suggesting this locus may represent a hot spot for insertions. Partial sequencing across the junction between EF3216 and EF3230 suggested that several of the non-CC2 strains carry genes homologous to OG1RF_0214 to -18 in this locus (results not shown).

Mobile DNA constitutes a substantial fraction of the *E. faecalis *V583 genome and transfer of MGEs and transposons thus plays an important role in the evolution of *E. faecalis *genomes [32]. The large pool of mobile elements also represents an abundant source of pseudogenes, as indel events occurring within coding regions often render genes nonfunctional. To verify the expression of the CC2-enriched genes, we correlated the list of enriched genes with data from two transcriptional analyses performed in our laboratory with the same array as used in the CGH experiment described in present study ([30] and Solheim, unpublished work). Transcription was confirmed for all but fifteen of the CC2-enriched genes (results not shown), thus validating the expression of these reading frames. The fifteen genes, for which no transcripts were detected, were mainly located within *efaB5 *and *phage04*.

**Figure 2:**
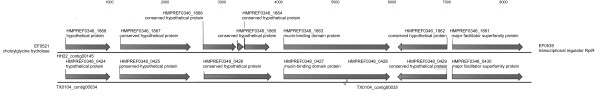
**Schematic representation of a putative non-V583 CC2-enriched gene cluster, as annotated in the *Enterococcus faecalis *HH22 and TX0104 draft genomes (GenBank accession numbers **ACIX00000000** and **ACGL00000000**, respectively)**. The EF-numbers of flanking genes indicate the insert site location compared to the *E. faecalis *V583 pathogenicity island.

A constraint of the comparative genomic analyses presented here, is that the comparison of gene content is based on a single reference strain only (V583). To compensate, we conducted a CC2 pangenome analysis with the draft genomes of CC2-strains HH22 and TX0104 to identify putative CC2-enriched non-V583 genes. The pangenome analysis identified a total of 298 non-V583 ORFs in the HH22 and TX0104 (Additional file 4). Among these ORFs, one gene cluster was identified as particularly interesting (Fisher's exact; Additional file 4 and Figure 2). Notably, HMPREF0348_0426 in TX0104 represented the best BLAST hit for all the three ORFs HMPREF0364_1864 to -66 in HH22, suggesting discrepancy in annotation between the two strains. Sequencing across the gap between contig 00034 and contig 00035 in TX0104 confirmed that HMPREF0348_0427 and HMPREF0348_0428 represent the two respective ends of a gene homologous to HMPREF0346_1863 in HH22. (Additional file 5). The presence of the putative non-V583 CC2-enriched gene cluster among *E. faecalis *was further elucidated by PCR in our collection of strains (Additional file 3). Strains were screened for the presence of three individual genes (HMPREF0346_1861, HMPREF0346_1864 and HMPREF0346_1868) and the entire element, with primers hmpref0346_1868-F and hmpref0346_1861-R. Fisher's exact testing (*q *< 0.01) on the basis of the PCR data confirmed that the gene cluster was significantly enriched among CC2. Comparative sequence analysis of the flanking regions suggests that the gene cluster is located in the HH22 and TX0104 versions of the *E. faecalis *pathogenicity island [36]. Recently, a microarray-based assessment of PAI-content in a set of clinical *E. faecalis *isolates revealed high degree of variation within the island, and an evidently modular evolution of the PAI [37], which would be consistent with acquisition by an indel event of this locus in the PAI of TX0104, HH22 and other positive CC2-strains.

### CC2-enriched surface-related structures

Lepage et al. [38] have previously identified eight genes as potential markers for the V583/MMH594-lineage, of which all except one gene (EF2513) are found among the CC2-enriched genes in this study. Interestingly, several of these genes were later assigned to a recently classified family of surface proteins, with a C-terminal WxL domain, proposed to form multi-component complexes on the cell surface [39,40]. Siezen et al. [40] termed these genes cell-surface complex (*csc*) genes and postulated a role in carbon source acquisition. Independently, Brinster et al. [39] showed that WxL domains are involved in peptidoglycan-binding. A total of nine WxL protein-coding genes, divided into three clusters (EF2248 to -54, EF3153 to -55 and EF3248 to -53), were identified as putative CC2-enriched genes in the present study. Note that EF3153 to - 55 does not represent a complete *csc *gene cluster, as not all four *csc *gene families (*cscA *- *cscD*) are present in the cluster [40]. Interestingly, the OG1RF genome sequence revealed homologues loci encoding WxL-proteins corresponding to the gene clusters EF3153 to -55 and EF3248 to -53 in V583 (50-75% sequence identity) [24]. Such homologs may possibly explain the divergence observed between CC2 and non-CC2-strains in the present study. Indeed, BLAST analysis with the OG1RF sequences against the *E. faecalis *draft genomes suggested that the OG1RF_0209-10 and OG1RF_0224-25 are widely distributed among non-CC2 *E. faecalis*. Given the putative function in carbon metabolism, the observed sequence variation may be related to substrate specificity.

In addition to the WxL domain, EF2250 also encodes a domain characteristic for the internalin family [39]. Internalins are characterized by the presence of N-terminal leucine-rich repeats (LRRs). The best characterized bacterial LRR proteins are InlA and InlB from *Listeria monocytogenes*, known to trigger internalization by normally non-phagocytic cells [41]. Two internalin-like proteins were identified in *E. faecalis *V583 (EF2250 and *elrA *(EF2686)) [41,42]. Recently, Brinster et al. [42] presented evidence of that ElrA play a role in *E. faecalis *virulence, both in early intracellular survival in macrophages and by stimulating the host inflammatory response through IL-6 induction. Moreover, by quantitative real-time PCR Shepard and Gilmore [43] found that *elrA *was induced in *E. faecalis *MMH594 during exponential growth in serum and during both exponential and stationary growth in urine. Contradictory data have, however, been published for this and other strains using different methods [42,44]. Although it is tempting to speculate that EF2250 contributes to the interaction with the mammalian host, the role of internalins in *E. faecalis *pathogenesis is still not understood, and it may therefore be premature to extrapolate function solely on the basis of shared structural domains.

Glycosyl transferase family proteins are involved in the formation of a number of cell surface structures such as glycolipids, glycoproteins and polysaccharides [45]. *E. faecalis *is in possession of several capsular polysaccharides [46-48], with Cps and Epa being the best characterized. The *epa *(enterococcal polysaccharide antigen) cluster represents a rhamnose-containing polysaccharide which was originally identified in *E. faecalis *OG1RF [46]. The version of the *epa *cluster found in the V583 genome contains an insertion of four genes (EF2185 to -88) compared to OG1RF. This insertion appeared to be enriched among CC2. While EF 2185 and EF2187 encodes transposases of the IS256 family, the two remaining genes showed 100% identity to the two respective ends of a racemase domain protein in *E. faecalis *TX0104. Neighboring the *epa *cluster, two glycosyl transferases (EF2170 and EF2167) proposed as potential virulence factors [32], are part of a three operon locus (EF2172 to -66), possibly associated with lipopolysaccharide production. Five of the genes within this locus were also found to be enriched among CC2 in the present study.

Paulsen et al. [32] also listed other putative surface-exposed virulence genes, including a choline-binding protein (CBP; EF2662) and a putative MSCRAMM (microbial surface components recognizing adhesive matrix molecules; EF2347) that based on our analysis were found to be enriched in CC2. A role of CBPs in pneumococcal colonization and virulence has been established [49,50]. A number of putative MSCRAMMs have been identified in *E. faecalis *[51], however, only Ace (adhesion of collagen from *E. faecalis*; EF1099) has been characterized in detail: Ace was shown to mediate binding to collagen (type I and IV), dentin and laminin [52-54]. Lebreton et al. [55] recently presented evidence of an *in vivo *function of Ace in enterococcal infections other than involvement in the interaction with extracellular matrix. It was demonstrated that an *ace *deletion mutant was significantly impaired in virulence, both in an insect model and in an *in vivo*-*in vitro *murine macrophage models. The authors suggested that Ace may promote *E. faecalis *phagocytosis and that it may also be possible that Ace is involved in survival of enterococci inside phagocytic cells. Also the structurally related MSCRAMM, Acm, found in *E. faecium *was recently reported to contribute to the pathogenesis of this bacterium [56].

Mucins are high molecular weight glycoproteins expressed by a wide variety of epithelial cells, including those of the gastrointestinal tract, and located at the interface between the cell and the surrounding environment [57]. The binding of bacteria to mucins through mucin-binding domain proteins is thought to promote colonization [58]. Diversity in the carbohydrate side chains creates a significant heterogeneity among mucins of different origin (*e.g*. different organisms or body sites), facilitating bacterial attachment to epithelial cells [58]. The non-V583 CC2-enriched gene cluster identified through *in silico *analysis in the present study harboured an ORF (HMPREF0346_1863 and HMPREF0348_0427/HMPREF0348_0428 in HH22 and TX0104, respectively) with homology to known mucin-binding domain proteins.

## Conclusions

In conclusion, we have identified a set of genes that appear to be enriched among strains belonging to CC2. Since a significant proportion (9.1%; *p *= 0.036, Fisher's exact test) of these genes code for proteins associated with cell surface structures, absence of or divergence in these loci may lead to antigenic variation. Indeed, both MSCRAMMs and internalins have been identified as potential antigens of *E. faecalis *or other Gram-positive bacteria [59-61]. It is noteworthy that the genes encoding any of the established enterococcal virulence factors were not among the CC2-enriched genes. Surface structures that promote adhesion of pathogenic bacteria to human tissue are also promising targets for creation of effective vaccines. However, functional studies of the individual CC2-enriched genes are required in order to distinguish their implications in enterococcal virulence.

## Methods

### Bacterial strain and growth conditions

Bacterial strains used in this study are listed in Table 1. *E. faecalis *strains were grown overnight (ON) in brain heart infusion broth (BHI; Oxoid) at 37° without shaking. All the strains have previously been sequence typed by the MLST scheme proposed by Ruiz-Garbajosa et al. [26].

### Comparative genomic hybridization

#### Microarrays

The microarray used in this work has been described previously [27]. The microarray design has been deposited in the ArrayExpress database with the accession number A-MEXP-1069 and A-MEXP-1765.

#### DNA isolation

Genomic DNA was isolated by using the FP120 FastPrep bead-beater (BIO101/Savent) and the QiaPrep MiniPrep kit (Qiagen) as previously described [27].

### Fluorescent labeling and hybridization

Fifteen hospital-associated *E. faecalis *strains were selected for CGH based on their representation of MLST sequence types (STs) belonging to major CCs and potential HiRECCs, with a special focus on CC2, and their variety of geographical origins within Europe. Genomic DNA was labeled and purified with the BioPrime Array CGH Genomic labeling System (Invitrogen) and Cyanine Smart Pack dUTP (PerkinElmer Life Sciences), according to the manufacturer's protocol. Purified samples were then dried, prior to resuspension in 140 μl hybridization solution (5 × SSC, 0.1% (w/v) SDS, 1.0% (w/v) bovine serum albumin, 50% (v/v) formamide and 0.01% (w/v) single-stranded salmon sperm DNA) and hybridized for 16 h at 42°C to the *E. faecalis *oligonucleotide array in a Tecan HS 400 pro hybridization station (Tecan). Arrays were washed twice at 42°C with 2 × SSC + 0.2% SDS, and twice at 23°C with 2 × SSC, followed by washes at 23°C with 1) 0.2 × SSC and 2) H_2_O. Two replicate hybridizations (dye-swap) were performed for each test strain. Hybridized arrays were scanned at wavelengths of 532 nm (Cy3) and 635 nm (Cy5) with a Tecan scanner LS (Tecan). Fluorescent intensities and spot morphologies were analyzed using GenePix Pro 6.0 (Molecular Devices), and spots were excluded based on slide or morphology abnormalities. All water used for the various steps of the hybridization and for preparation of solutions was filtered (0.2 μM) MilliQ dH_2_0.

### Data analysis

Standard methods in the LIMMA package [62] in R http://www.r-project.org/, available from the Bioconductor http://www.bioconductor.org were employed for preprocessing and normalization. Within-array normalization was first conducted by subtracting the median from the log-ratios for each array. A standard loess-normalization was then performed, where smoothing was based only on spots with abs(log-ratio) < 2.0 to avoid biases due to extreme skewness in the log-ratio distribution. For the determination of present and divergent genes a method that predicts sequence identity based on array signals was used, as described by Snipen et al. [63]. A threshold of 0.75 was used in order to obtain a categorical response of presence or divergence, *i. e*. genes with Sb-value > 0.75 were classified as present, while genes with Sb-value < 0.75 were classified as divergent. Genes with Sb-value = 0.75 remained unclassified. All genes were tested for significant enrichment among the CC2-strains by using the Fisher's exact test.

### Microarray data accession number

The microarray data have been deposited in the ArrayExpress database with the series accession number E-TABM-905.

### Polymerase chain reaction

The presence of selected genes was verified by means of polymerase chain reactions (PCR). A similar approach was also applied to investigate the presence of selected mobile genetic elements (MGEs). Primers targeting the genes flanking the MGEs were combined with internal primers to monitor the presence of the junctions on either side of each MGE. PCR was carried out in 20 μl reaction volumes containing 1× buffer, 250 μM of each deoxynucleotide triphosphate and 1 U DyNAZyme II polymerase (Finnzymes). The reaction conditions included an initial denaturation step at 95°C and 35 cycles of 95°C for 30 s, 56-60°C for 30 s and 72°C for 1-5 min, followed by a final extension step at 72°C for 7 min. The primers used in this study are listed in Table 2.

**Table 2:** Primers used in this study.

Target gene	Primer sequences (5' → 3')	Amplicon size (bp)	Application
*ef1415*	F:TGTTGCGGTTTCTGCATTAG	2818	PCR on junction between EF1415 and EF1417
*ef1417*	R:GCATCTCGATAGACAATTCG		PCR on junction between EF1415 and EF1417
*ef1489*	F:GAATCGAACTAGCATTTTTGGG	465	PCR on junction between EF1489 and EF1490
*ef1490*	R:ATGGAACGAACCATTGGAAA		PCR on junction between EF1489 and EF1490
*ef1843*	F:GGAGCCGTTAGACAGACAGC	2457	PCR on junction between EF1843 and EF1847
*ef1847*	R:GCTTGCTTTACAGCCTCAAGA		PCR on junction between EF1843 and EF1847
*ef1895*	F:GCACAACAAATTTCAATTCCA	4573	PCR on junction between EF1895 and EF1898
*ef1898*	R:ATTGAAGTGGTTCGCTACGG		PCR on junction between EF1895 and EF1898
*ef2239*	F:AACTGCTGTCAAGCGTAGCA	1252	PCR on junction between EF2239 and EF2240
*ef2240*	R:TGTGGCATTTTGGACTGTTG		PCR on junction between EF2239 and EF2240
*ef2350*	F:ATAACTGAGTGATTTTCACAATTGC	654	PCR on junction between EF2350 and EF2352
*ef2352*	R:GATCCGTGGAAGTTCCTCAA		PCR on junction between EF2350 and EF2352
*ef3216*	F:TCGGCGTTGAAGACTATGAA	-	Sequencing of junction between EF3216 and EF3230
*ef3217*	F:ATTGGGAATGACGGCTACAC R:TTGCGTATTTCGCAGCATAA	499	PCR
*ef3218*	F:TCGCGTAGTAGGAGCAATCA R:TTTTGTTCAGTTCCCACACCT	396	PCR
*ef3220*	F:AGCTTTTGGCGAAGGAGATT R:TTTATTGCGGGTTCCTCAGT	495	PCR
*ef3221*	F:TGAACGAAAATGAAGGTGGT R:TCATCAATCTCCAACGCATC	196	PCR
*ef3222*	F:CAAAGAAGAATCAGCCGATTAAA R:ATATTTGGGCATTTGCATGG	183	PCR
*ef3223*	F:AATTGGGAAAAAGGGGTCAG R:TTCGTGATCTGCTTGTTGTTCT	501	PCR
*ef3224*	F:GTTGGGCTGGACGTATGAAT R:TGTGGCTTTATAGGCTGTAGCA	214	PCR
*ef3225*	F:ATTACTTCACCGCCCATGAC R:CGCTGGAAGTCTGCTCTTG	474	PCR
*ef3226*	F:GATGATTTAACCGCACAAGGA R:TTTTTATTTCGAGCGGATGC	499	PCR
*ef3227*	F:ACAGGAAGCCATTCACAAACT R:CTGATTCGTGGAAGTCCAACT	162	PCR
*ef3230*	R:TCCTGACTTCCGTTCTGCTT	-	Sequencing of junction between EF3216 and EF3230
*hmpref0346_1861*	F:CGAGTTAGAGGAAGCGTTGG	630	PCR
	R:CCAGACAATTTGGGCGTACT		
*hmpref0346_1864*	F:GAAATTTTCTGAAAGTGAAGACAAGA	299	PCR
	R:TGATTAGCAGTCACAACAGCAA		
*hmpref0346_1868*	F:TGTACACAAGCTACCCGGATT	538	PCR
	R:TTCCCACCTGCGTCTATTTT		
*hmpref0348_0427*	R:GAGACTTCAACCACTCCACAAAAACC	-	Sequencing of gap between contig00034-35 in TX0104
*hmpref0348_0428*	F:CCTGTAGAAGTATTGTCCATTTTAACGCTATC		Sequencing of gap between contig00034-35 in TX0104

### Validation of microarray data by sequencing

Sequencing was performed using the ABI Prism Big dye Cycle Sequencing Ready Reaction kit (Applied Biosystems) in an ABI PrismTM 3100 Genetic Analyzer and primers listed in Table 2.

### *In silico *comparison of *E. faecalis *draft genomes

Whole genome blast comparison against the V583 reference genome was conducted for 24 *E. faecalis *strains whose draft genomes were publicly available (GenBank accession numbers in parenthesis; Table 1): *E. faecalis *ARO1/DG (ACAK01000000); *E. faecalis *ATCC 4200 (ACAG01000000); *E. faecalis *ATCC 29200 (ACOX00000000); *E. faecalis *CH188 (ACAV01000000); *E. faecalis *D6 (ACAT01000000); *E. faecalis *DS5 (ACAI01000000); *E. faecalis *E1Sol (ACAQ01000000); *E. faecalis *Fly1 (ACAR01000000): *E. faecalis *HIP11704 (ACAN01000000); *E. faecalis *HH22 (ACIX00000000); *E. faecalis *JH1 (ACAP01000000); *E. faecalis *Merz96 (ACAM01000000); *E. faecalis *OG1RF (ABPI01000001); *E. faecalis *R712 (ADDQ00000000); *E. faecalis *S613 (ADDP00000000); *E. faecalis *T1 (ACAD01000000); *E. faecalis *T2 (ACAE01000000); *E. faecalis *T3 (ACAF01000000); *E. faecalis *T8 (ACOC01000000); *E. faecalis *T11 (ACAU01000000); *E. faecalis *TuSoD ef11(ACOX00000000); *E. faecalis *TX0104 (ACGL00000000); *E. faecalis *TX1322 (ACGM00000000); *E. faecalis *X98 (ACAW01000000) [64,65], as follows: the annotated V583 genes were blasted (BLASTN) against each genome, and presence and divergence was predicted based on a score calculated as number of identical nucleotides divided by the length of the query gene. Genes obtaining a score >0.75 were predicted to be present.

### CC2 pangenome content analysis

Among the newly released *E. faecalis *draft genomes were two CC2-strains; HH22 and TX0104. In order to extend the list of CC2-enriched genes beyond V583, we conducted a BLAST search using the annotated genes of these two strains as queries against the full genome sequences of the other draft genomes. Again, a cutoff of 75% identity to the query was used to distinguish present from divergent genes.

## Authors' contributions

MS conceived and designed the study, carried out the experimental work, analyzed the data, assisted in the bioinformatic analysis and drafted the manuscript. MCB performed the experimental work and assisted in critical review of the manuscript. LS contributed analysis tools, performed the statistical and bioinformatic analyses and assisted in the critical review of the manuscript. RJLW conceived and designed the study, contributed material and assisted in critical review of the manuscript. IFN conceived the study, contributed material and assisted in critical review of the manuscript. DAB participated in the design and coordination of the study, performed bioinformatic analysis and helped to draft the manuscript. All authors read and approved the final manuscript.

## Supplementary Material

Additional file 1 **BLAST comparison of *E. faecalis *genomes**. Data from BLAST comparison of 24 *E. faecalis *draft genomes with the annotated genes of strain V583. Click here for file

Additional file 2 **V583 genes which were identified as significantly enriched among CC2-strains in the present study**. A list of V583 genes which were identified as significantly enriched among CC2-strains in the present study. Click here for file

Additional file 3 **PCR screening**. An overview of results from PCR screening of a collection of *E. faecalis *isolates. Click here for file

Additional file 4 **Enrichment analysis of CC6 non-V583 genes by Fisher's exact test**. An overview of the presence non-V583 genes in 24 *E. faecalis *draft genomes CC6 including data from enrichment analysis by Fisher's exact test. Click here for file

Additional file 5 **Amino acid alignment of HMPREF0346_1863 in *Enterococcus faecalis *HH22 and its homologue in *E. faecalis *TX0104**. An amino acid alignment of HMPREF0346_1863 in *Enterococcus faecalis *HH22 and its homologue in *E. faecalis *TX0104. Click here for file
