# Supplementary material for: Comparative genomic analysis reveals significant enrichment of mobile genetic elements and genes encoding surface structure-proteins in hospital-associated clonal complex 2 Enterococcus faecalis
Source: BMC Microbiol. 2011 Jan 4;11:3. doi: 10.1186/1471-2180-11-3 (PMC3022643; doi:10.1186/1471-2180-11-3)
Supplement: Additional file 2 — V583 genes which were identified as significantly enriched among CC2-strains in the present study. A list of V583 genes which were identified as significantly enriched among CC2-strains in the present study. [file 1471-2180-11-3-S2.DOC]

**Additional file 2.** V583 genes which were identified as significantly enriched among CC2-strains in the present study. The statistical analysis was based on a compiled data set including comparative genomic hybridization and genomic BLAST data from a total of 64 *E. faecalis* isolates. CC; clonal complex, FDR; false discovery rate, p01; phage01, p03; phage03, p04; phage04, p06;phage06 and PAI; pathogenicity island.

| **ORF** | **Gene product** | **Mobile genetic element** | **CC2-strains present** | | **Non-CC2 strains present** | | ***q*-value (FDR)** |
| --- | --- | --- | --- | --- | --- | --- | --- |
|  |  |  | **(n=10)** | **(%)** | **(n=54)** | **(%)** |  |
| EF0052 | Hypothetical protein |  | 8 | (80) | 3 | (6) | 4E-05 |
| EF0053 | DNA polymerase III, epsilon subunit |  | 8 | (80) | 2 | (4) | 1E-05 |
| EF0054 | Hypothetical protein |  | 8 | (80) | 2 | (4) | 1E-05 |
| EF0112 | Conserved domain protein |  | 8 | (80) | 4 | (7) | 8E-05 |
| EF0113 | Hypothetical protein |  | 8 | (80) | 4 | (7) | 8E-05 |
| EF0126 | Conserved hypothetical protein |  | 8 | (80) | 5 | (9) | 2E-04 |
| EF0315 | Conserved hypothetical protein | *p01* | 4 | (40) | 1 | (2) | 9E-03 |
| EF0316 | Hypothetical protein | *p01* | 4 | (40) | 1 | (2) | 9E-03 |
| EF0325 | DNA polymerase, putative | *p01* | 5 | (50) | 3 | (6) | 9E-03 |
| EF0329 | Conserved hypothetical protein | *p01* | 5 | (50) | 3 | (6) | 9E-03 |
| EF0330 | SNF2 domain protein | *p01* | 4 | (40) | 1 | (2) | 9E-03 |
| EF0500 | Conserved hypothetical protein | PAI | 10 | (100) | 23 | (43) | 6E-03 |
| EF0510 | Single-strand binding protein | PAI | 10 | (100) | 25 | (46) | 9E-03 |
| EF0568 | Potassium-transporting ATPase, subunit B | PAI | 7 | (70) | 9 | (17) | 9E-03 |
| EF0569 | Potassium-transporting ATPase, subunit C | PAI | 7 | (70) | 9 | (17) | 9E-03 |
| EF0570 | Sensor histidine kinase KdpD | PAI | 7 | (70) | 9 | (17) | 9E-03 |
| EF0574 | Hypothetical protein | PAI | 7 | (70) | 9 | (17) | 9E-03 |
| EF0594 | Hypothetical protein | PAI | 7 | (70) | 9 | (17) | 9E-03 |
| EF0967 | Conserved domain protein |  | 10 | (100) | 18 | (33) | 9E-04 |
| EF1031 | Phosphorylase family protein |  | 10 | (100) | 19 | (35) | 1E-03 |
| EF1329 | HesA/MoeB/ThiF family protein |  | 10 | (100) | 22 | (41) | 6E-03 |
| EF1330 | Hypothetical protein |  | 10 | (100) | 20 | (37) | 2E-03 |
| EF1331 | ABC transporter, ATP-binding protein |  | 10 | (100) | 20 | (37) | 2E-03 |
| EF1332 | Membrane protein, putative |  | 10 | (100) | 20 | (37) | 2E-03 |
| EF1333 | ABC transporter, ATP-binding protein |  | 10 | (100) | 20 | (37) | 2E-03 |
| EF1334 | AgrC domain protein |  | 10 | (100) | 20 | (37) | 2E-03 |
| EF1335 | Sensor histidine kinase, putative |  | 10 | (100) | 20 | (37) | 2E-03 |
| EF1336 | Response regulator |  | 10 | (100) | 21 | (39) | 2E-03 |
| EF1417 | Site-specific recombinase, phage integrase family | *p03* | 8 | (80) | 6 | (11) | 3E-04 |
| EF1418 | Hypothetical protein | *p03* | 7 | (70) | 8 | (15) | 6E-03 |
| EF1419 | Conserved hypothetical protein | *p03* | 6 | (60) | 2 | (4) | 8E-04 |
| EF1423 | Transcriptional regulator, Cro/CI family | *p03* | 5 | (50) | 2 | (4) | 5E-03 |
| EF1424 | Hypothetical protein | *p03* | 8 | (80) | 6 | (11) | 3E-04 |
| EF1425 | Hypothetical protein | *p03* | 8 | (80) | 6 | (11) | 3E-04 |
| EF1426 | VrlI protein, putative | *p03* | 7 | (70) | 6 | (11) | 2E-03 |
| EF1428 | Hypothetical protein | *p03* | 6 | (60) | 5 | (9) | 6E-03 |
| EF1429 | Hypothetical protein | *p03* | 7 | (70) | 7 | (13) | 4E-03 |
| EF1430 | Conserved hypothetical protein | *p03* | 8 | (80) | 6 | (11) | 3E-04 |
| EF1433 | Conserved hypothetical protein | *p03* | 8 | (80) | 6 | (11) | 3E-04 |
| EF1434 | DnaD domain protein | *p03* | 8 | (80) | 6 | (11) | 3E-04 |
| EF1435 | Recombination protein U, putative | *p03* | 8 | (80) | 6 | (11) | 3E-04 |
| EF1436 | Hypothetical protein | *p03* | 8 | (80) | 6 | (11) | 3E-04 |
| EF1443 | Conserved hypothetical protein | *p03* | 7 | (70) | 5 | (9) | 1E-03 |
| EF1445 | Replicase domain protein | *p03* | 8 | (80) | 7 | (13) | 6E-04 |
| EF1446 | Hypothetical protein | *p03* | 8 | (80) | 9 | (17) | 2E-03 |
| EF1447 | Conserved hypothetical protein | *p03* | 8 | (80) | 5 | (9) | 2E-04 |
| EF1448 | Hypothetical protein | *p03* | 8 | (80) | 6 | (11) | 3E-04 |
| EF1449 | Conserved hypothetical protein | *p03* | 8 | (80) | 6 | (11) | 3E-04 |
| EF1450 | Positive control factor, putative | *p03* | 8 | (80) | 6 | (11) | 3E-04 |
| EF1455 | Terminase, large subunit, putative | *p03* | 8 | (80) | 6 | (11) | 3E-04 |
| EF1456 | Conserved hypothetical protein TIGR01555 | *p03* | 7 | (70) | 5 | (9) | 1E-03 |
| EF1457 | Minor head protein | *p03* | 8 | (80) | 5 | (9) | 2E-04 |
| EF1458 | Hypothetical protein | *p03* | 8 | (80) | 5 | (9) | 2E-04 |
| EF1459 | Conserved hypothetical protein | *p03* | 8 | (80) | 7 | (13) | 6E-04 |
| EF1460 | LysM domain protein | *p03* | 8 | (80) | 5 | (9) | 2E-04 |
| EF1461 | Conserved hypothetical protein | *p03* | 8 | (80) | 6 | (11) | 3E-04 |
| EF1462 | Conserved hypothetical protein | *p03* | 7 | (70) | 8 | (15) | 6E-03 |
| EF1463 | Hypothetical protein | *p03* | 7 | (70) | 6 | (11) | 2E-03 |
| EF1464 | Conserved hypothetical protein | *p03* | 8 | (80) | 5 | (9) | 2E-04 |
| EF1465 | Conserved hypothetical protein | *p03* | 8 | (80) | 5 | (9) | 2E-04 |
| EF1466 | Conserved hypothetical protein | *p03* | 7 | (70) | 6 | (11) | 2E-03 |
| EF1467 | Conserved hypothetical protein | *p03* | 7 | (70) | 6 | (11) | 2E-03 |
| EF1469 | Conserved hypothetical protein | *p03* | 8 | (80) | 6 | (11) | 3E-04 |
| EF1470 | Conserved hypothetical protein | *p03* | 8 | (80) | 6 | (11) | 3E-04 |
| EF1471 | Conserved hypothetical protein | *p03* | 8 | (80) | 8 | (15) | 1E-03 |
| EF1472 | Conserved hypothetical protein | *p03* | 8 | (80) | 6 | (11) | 3E-04 |
| EF1473 | Conserved hypothetical protein | *p03* | 8 | (80) | 6 | (11) | 3E-04 |
| EF1474 | LysM domain protein | *p03* | 8 | (80) | 6 | (11) | 3E-04 |
| EF1475 | Conserved hypothetical protein | *p03* | 8 | (80) | 6 | (11) | 3E-04 |
| EF1476 | Conserved hypothetical protein | *p03* | 8 | (80) | 6 | (11) | 3E-04 |
| EF1477 | Conserved hypothetical protein | *p03* | 8 | (80) | 6 | (11) | 3E-04 |
| EF1478 | Conserved hypothetical protein | *p03* | 8 | (80) | 7 | (13) | 6E-04 |
| EF1479 | Conserved hypothetical protein | *p03* | 8 | (80) | 6 | (11) | 3E-04 |
| EF1480 | Conserved hypothetical protein | *p03* | 8 | (80) | 7 | (13) | 6E-04 |
| EF1481 | Hypothetical protein | *p03* | 7 | (70) | 5 | (9) | 1E-03 |
| EF1482 | Hypothetical protein | *p03* | 8 | (80) | 6 | (11) | 3E-04 |
| EF1483 | Conserved hypothetical protein | *p03* | 8 | (80) | 8 | (15) | 1E-03 |
| EF1484 | Conserved hypothetical protein | *p03* | 8 | (80) | 6 | (11) | 3E-04 |
| EF1485 | Conserved hypothetical protein | *p03* | 8 | (80) | 6 | (11) | 3E-04 |
| EF1589 | Acetyltransferase, GNAT family |  | 10 | (100) | 25 | (46) | 9E-03 |
| EF1825 | Conserved domain protein |  | 10 | (100) | 23 | (43) | 6E-03 |
| EF1826 | Alcohol dehydrogenase, zinc-containing |  | 10 | (100) | 22 | (41) | 6E-03 |
| EF1827 | Conserved hypothetical protein |  | 10 | (100) | 18 | (33) | 9E-04 |
| EF1828 | Glycerol uptake facilitator protein, putative |  | 10 | (100) | 18 | (33) | 9E-04 |
| EF1829 | PTS system, IID component |  | 10 | (100) | 18 | (33) | 9E-04 |
| EF1830 | PTS system, IIC component |  | 10 | (100) | 18 | (33) | 9E-04 |
| EF1833 | PTS system component, authentic frameshift |  | 10 | (100) | 22 | (41) | 6E-03 |
| EF1834 | galactose-6-phosphate isomerase, LacB subunit |  | 10 | (100) | 22 | (41) | 6E-03 |
| EF1835 | galactose-6-phosphate isomerase, LacA subunit |  | 10 | (100) | 22 | (41) | 6E-03 |
| EF1836 | PTS system, IIA component, putative |  | 10 | (100) | 22 | (41) | 6E-03 |
| EF1837 | PTS system, IIB component, putative |  | 10 | (100) | 22 | (41) | 6E-03 |
| EF1838 | PTS system, IIC component |  | 10 | (100) | 22 | (41) | 6E-03 |
| EF1844 | Hypothetical protein |  | 10 | (100) | 21 | (39) | 2E-03 |
| EF1847 | Site-specific recombinase, phage integrase family | *efaB5* | 10 | (100) | 20 | (37) | 2E-03 |
| EF1848 | Hypothetical protein | *efaB5* | 10 | (100) | 18 | (33) | 9E-04 |
| EF1849 | Conserved hypothetical protein | *efaB5* | 10 | (100) | 22 | (41) | 6E-03 |
| EF1851 | Glycosyl hydrolase, family 35 | *efaB5* | 10 | (100) | 21 | (39) | 2E-03 |
| EF1853 | Hypothetical protein | *efaB5* | 10 | (100) | 20 | (37) | 2E-03 |
| EF1855 | Transposase, IS256 family | *efaB5* | 10 | (100) | 20 | (37) | 2E-03 |
| EF1858 | Aspartate 1-decarboxylase | *efaB5* | 10 | (100) | 20 | (37) | 2E-03 |
| EF1859 | Pantoate--beta-alanine ligase | *efaB5* | 10 | (100) | 20 | (37) | 2E-03 |
| EF1860 | 3-methyl-2-oxobutanoate hydroxymethyltransferase | *efaB5* | 10 | (100) | 20 | (37) | 2E-03 |
| EF1861 | Conserved domain protein | *efaB5* | 10 | (100) | 20 | (37) | 2E-03 |
| EF1863 | Sensor histidine kinase | *efaB5* | 10 | (100) | 20 | (37) | 2E-03 |
| EF1864 | DNA-binding response regulator | *efaB5* | 10 | (100) | 20 | (37) | 2E-03 |
| EF1867 | Permease, putative | *efaB5* | 10 | (100) | 20 | (37) | 2E-03 |
| EF1868 | ABC transporter, ATP-binding protein | *efaB5* | 10 | (100) | 21 | (39) | 2E-03 |
| EF1869 | Permease, putative | *efaB5* | 10 | (100) | 20 | (37) | 2E-03 |
| EF1871 | Thioredoxin family protein | *efaB5* | 10 | (100) | 23 | (43) | 6E-03 |
| EF1872 | Conserved domain protein | *efaB5* | 10 | (100) | 22 | (41) | 6E-03 |
| EF1875 | Conserved hypothetical protein | *efaB5* | 10 | (100) | 21 | (39) | 2E-03 |
| EF1876 | Lipoprotein, NLP/P60 family | *efaB5* | 10 | (100) | 20 | (37) | 2E-03 |
| EF1877 | Membrane protein, putative | *efaB5* | 10 | (100) | 24 | (44) | 7E-03 |
| EF1879 | Conserved hypothetical protein | *efaB5* | 10 | (100) | 21 | (39) | 2E-03 |
| EF1880 | Hypothetical protein | *efaB5* | 10 | (100) | 20 | (37) | 2E-03 |
| EF1881 | Conserved domain protein | *efaB5* | 10 | (100) | 20 | (37) | 2E-03 |
| EF1882 | Conserved hypothetical protein | *efaB5* | 10 | (100) | 20 | (37) | 2E-03 |
| EF1884 | Transposase, IS116/IS110/IS902 family | *efaB5* | 10 | (100) | 21 | (39) | 2E-03 |
| EF1885 | Hypothetical protein | *efaB5* | 10 | (100) | 24 | (44) | 7E-03 |
| EF1886 | Transcriptional regulator, Cro/CI family | *efaB5* | 10 | (100) | 22 | (41) | 6E-03 |
| EF1887 | Conserved hypothetical protein | *efaB5* | 10 | (100) | 20 | (37) | 2E-03 |
| EF1888 | Hypothetical protein | *efaB5* | 10 | (100) | 22 | (41) | 6E-03 |
| EF1889 | Conserved domain protein | *efaB5* | 10 | (100) | 25 | (46) | 9E-03 |
| EF1892 | FtsK/SpoIIIE family protein | *efaB5* | 10 | (100) | 24 | (44) | 7E-03 |
| EF1894 | Conserved hypothetical protein | *efaB5* | 10 | (100) | 21 | (39) | 2E-03 |
| EF1895 | Conserved hypothetical protein | *efaB5* | 10 | (100) | 20 | (37) | 2E-03 |
| EF1897 | Hypothetical protein |  | 10 | (100) | 23 | (43) | 6E-03 |
| EF1993 | holin | *p04* | 10 | (100) | 23 | (43) | 6E-03 |
| EF1994 | Conserved hypothetical protein | *p04* | 10 | (100) | 25 | (46) | 9E-03 |
| EF1995 | Hypothetical protein | *p04* | 10 | (100) | 24 | (44) | 7E-03 |
| EF2006 | Hypothetical protein | *p04* | 10 | (100) | 24 | (44) | 7E-03 |
| EF2012 | Conserved hypothetical protein | *p04* | 10 | (100) | 23 | (43) | 6E-03 |
| EF2014 | Coenzyme F420 hydrogenase domain protein | *p04* | 10 | (100) | 12 | (22) | 8E-05 |
| EF2015 | Minor head protein, putative | *p04* | 9 | (90) | 16 | (30) | 4E-03 |
| EF2017 | Terminase, large subunit, putative | *p04* | 9 | (90) | 10 | (19) | 3E-04 |
| EF2018 | Conserved hypothetical protein | *p04* | 10 | (100) | 5 | (9) | 7E-07 |
| EF2019 | Hypothetical protein | *p04* | 10 | (100) | 4 | (7) | 3E-07 |
| EF2020 | Hypothetical protein | *p04* | 10 | (100) | 4 | (7) | 3E-07 |
| EF2022 | Conserved domain protein | *p04* | 10 | (100) | 5 | (9) | 7E-07 |
| EF2027 | Conserved hypothetical protein | *p04* | 10 | (100) | 20 | (37) | 2E-03 |
| EF2028 | Hypothetical protein | *p04* | 9 | (90) | 17 | (31) | 6E-03 |
| EF2031 | Conserved hypothetical protein | *p04* | 10 | (100) | 23 | (43) | 6E-03 |
| EF2032 | Hypothetical protein | *p04* | 10 | (100) | 23 | (43) | 6E-03 |
| EF2033 | Hypothetical protein | *p04* | 10 | (100) | 14 | (26) | 2E-04 |
| EF2034 | Hypothetical protein | *p04* | 10 | (100) | 21 | (39) | 2E-03 |
| EF2039 | Conserved hypothetical protein | *p04* | 10 | (100) | 11 | (20) | 5E-05 |
| EF2040 | transcriptional regulator, Cro/CI family | *p04* | 9 | (90) | 7 | (13) | 8E-05 |
| EF2042 | Conserved domain protein | *p04* | 10 | (100) | 8 | (15) | 8E-06 |
| EF2164 | Membrane protein, putative |  | 10 | (100) | 14 | (26) | 2E-04 |
| EF2166 | Membrane protein, putative |  | 10 | (100) | 16 | (30) | 4E-04 |
| EF2167 | Glycosyl transferase, group 2 family protein |  | 10 | (100) | 12 | (22) | 8E-05 |
| EF2168 | LicD1 protein, putative |  | 10 | (100) | 14 | (26) | 2E-04 |
| EF2169 | Membrane protein, putative |  | 10 | (100) | 12 | (22) | 8E-05 |
| EF2170 | Glycosyl transferase, group 2 family protein |  | 10 | (100) | 23 | (43) | 6E-03 |
| EF2174 | Conserved domain protein |  | 10 | (100) | 23 | (43) | 6E-03 |
| EF2186 | Conserved domain protein |  | 10 | (100) | 16 | (30) | 4E-04 |
| EF2188 | Racemase domain protein |  | 10 | (100) | 18 | (33) | 9E-04 |
| EF2240 | Site-specific recombinase, phage integrase family | *vanB*-ass. phage | 10 | (100) | 8 | (15) | 8E-06 |
| EF2241 | Conserved hypothetical protein | *vanB*-ass. phage | 10 | (100) | 7 | (13) | 4E-06 |
| EF2243 | Conserved hypothetical protein | *vanB*-ass. phage | 10 | (100) | 4 | (7) | 3E-07 |
| EF2244 | Conserved hypothetical protein | *vanB*-ass. phage | 10 | (100) | 5 | (9) | 8E-07 |
| EF2245 | Conserved hypothetical protein | *vanB*-ass. phage | 10 | (100) | 4 | (7) | 3E-07 |
| EF2247 | Transcriptional regulator | *vanB*-ass. phage | 10 | (100) | 4 | (7) | 3E-07 |
| EF2248 | Hypothetical protein | *vanB*-ass. phage | 8 | (80) | 4 | (7) | 8E-05 |
| EF2249 | Hypothetical protein | *vanB*-ass. phage | 10 | (100) | 4 | (7) | 3E-07 |
| EF2250 | Conserved domain protein | *vanB*-ass. phage | 10 | (100) | 4 | (7) | 3E-07 |
| EF2251 | Hypothetical protein | *vanB*-ass. phage | 10 | (100) | 4 | (7) | 3E-07 |
| EF2252 | Hypothetical protein | *vanB*-ass. phage | 10 | (100) | 4 | (7) | 3E-07 |
| EF2253 | Conserved hypothetical protein | *vanB*-ass. phage | 10 | (100) | 4 | (7) | 3E-07 |
| EF2254 | Conserved hypothetical protein | *vanB*-ass. phage | 10 | (100) | 4 | (7) | 3E-07 |
| EF2255 | Site-specific recombinase, phage integrase family | *vanB*-ass. phage | 10 | (100) | 4 | (7) | 3E-07 |
| EF2257 | PTS system, IIC component, putative | *vanB*-ass. phage | 10 | (100) | 4 | (7) | 3E-07 |
| EF2258 | Conserved domain protein | *vanB*-ass. phage | 10 | (100) | 4 | (7) | 3E-07 |
| EF2259 | Phosphosugar-binding transcriptional regulator | *vanB*-ass. phage | 10 | (100) | 6 | (11) | 2E-06 |
| EF2260 | Conserved hypothetical protein | *vanB*-ass. phage | 10 | (100) | 6 | (11) | 2E-06 |
| EF2261 | Hypothetical protein | *vanB*-ass. phage | 10 | (100) | 4 | (7) | 3E-07 |
| EF2262 | Hypothetical protein | *vanB*-ass. phage | 10 | (100) | 4 | (7) | 3E-07 |
| EF2263 | Gluconate 5-dehydrogenase, putative | *vanB*-ass. phage | 10 | (100) | 5 | (9) | 7E-07 |
| EF2264 | 4-deoxy-l-threo-5-hexosulose-uronate ketol-isomerase | *vanB*-ass. phage | 10 | (100) | 25 | (46) | 9E-03 |
| EF2265 | Carbohydrate kinase, pfkB family | *vanB*-ass. phage | 10 | (100) | 4 | (7) | 3E-07 |
| EF2266 | 2-dehydro-3-deoxyphosphogluconate aldolase/4-hydroxy-2-oxoglutarate aldolase, putative | *vanB*-ass. phage | 10 | (100) | 4 | (7) | 3E-07 |
| EF2267 | PTS system, IIA component | *vanB*-ass. phage | 10 | (100) | 6 | (11) | 2E-06 |
| EF2268 | Conserved hypothetical protein | *vanB*-ass. phage | 10 | (100) | 4 | (7) | 3E-07 |
| EF2269 | PTS system, IID component | *vanB*-ass. phage | 10 | (100) | 13 | 24 | 1E-04 |
| EF2270 | PTS system, IIC component | *vanB*-ass. phage | 10 | (100) | 4 | (7) | 3E-07 |
| EF2271 | PTS system, IIB component | *vanB*-ass. phage | 10 | (100) | 4 | (7) | 3E-07 |
| EF2272 | Glucuronyl hydrolase, putative | *vanB*-ass. phage | 10 | (100) | 4 | (7) | 3E-07 |
| EF2273 | Transcriptional regulator, GntR family | *vanB*-ass. phage | 10 | (100) | 4 | (7) | 3E-07 |
| EF2275 | Hypothetical protein | *vanB*-ass. phage | 10 | (100) | 4 | (7) | 3E-07 |
| EF2276 | Hypothetical protein | *vanB*-ass. phage | 10 | (100) | 4 | (7) | 3E-07 |
| EF2277 | Conserved hypothetical protein | *vanB*-ass. phage | 10 | (100) | 5 | (9) | 7E-07 |
| EF2278 | Lipoprotein, NLP/P60 family | *vanB*-ass. phage | 10 | (100) | 12 | (22) | 8E-05 |
| EF2279 | Membrane protein, putative | *vanB*-ass. phage | 10 | (100) | 4 | (7) | 3E-07 |
| EF2280 | Conserved hypothetical protein | *vanB*-ass. phage | 8 | (80) | 12 | (22) | 6E-03 |
| EF2281 | Conserved hypothetical protein | *vanB*-ass. phage | 10 | (100) | 14 | (26) | 2E-04 |
| EF2282 | Conserved domain protein | *vanB*-ass. phage | 10 | (100) | 6 | (11) | 2E-06 |
| EF2335 | Conserved hypothetical protein | *vanB*-ass. phage | 10 | (100) | 12 | (22) | 8E-05 |
| EF2337 | Hypothetical protein | *vanB*-ass. phage | 10 | (100) | 19 | (35) | 1E-03 |
| EF2338 | Transcriptional regulator, Cro/CI family | *vanB*-ass. phage | 9 | (90) | 14 | (26) | 2E-03 |
| EF2339 | Hypothetical protein | *vanB*-ass. phage | 10 | (100) | 4 | (7) | 3E-07 |
| EF2340 | C-5 cytosine-specific DNA methylase | *vanB*-ass. phage | 10 | (100) | 4 | (7) | 3E-07 |
| EF2341 | Hypothetical protein | *vanB*-ass. phage | 10 | (100) | 5 | (9) | 7E-07 |
| EF2342 | Hypothetical protein | *vanB*-ass. phage | 10 | (100) | 4 | (7) | 3E-07 |
| EF2343 | FtsK/SpoIIIE family protein | *vanB*-ass. phage | 10 | (100) | 4 | (7) | 3E-07 |
| EF2344 | Hypothetical protein | *vanB*-ass. phage | 10 | (100) | 13 | (24) | 1E-04 |
| EF2345 | Conserved hypothetical protein | *vanB*-ass. phage | 10 | (100) | 13 | (24) | 1E-04 |
| EF2346 | Conserved hypothetical protein | *vanB*-ass. phage | 10 | (100) | 14 | (26) | 2E-04 |
| EF2347 | Cell wall surface anchor family protein | *vanB*-ass. phage | 8 | (80) | 13 | (24) | 9E-03 |
| EF2348 | Hypothetical protein | *vanB*-ass. phage | 9 | (90) | 4 | (7) | 7E-06 |
| EF2349 | Hypothetical protein | *vanB*-ass. phage | 10 | (100) | 4 | (7) | 3E-07 |
| EF2350 | Transcriptional regulator, Cro/CI family | *vanB*-ass. phage | 10 | (100) | 4 | (7) | 3E-07 |
| EF2351 | Hypothetical protein | *vanB*-ass. phage | 10 | (100) | 4 | (7) | 3E-07 |
| EF2360 | Hypothetical protein |  | 10 | (100) | 22 | (41) | 6E-03 |
| EF2385 | Hypothetical protein |  | 10 | (100) | 4 | (7) | 3E-07 |
| EF2386 | Hypothetical protein |  | 10 | (100) | 4 | (7) | 3E-07 |
| EF2387 | chromosome partitioning ATPase, ParA family |  | 10 | (100) | 4 | (7) | 3E-07 |
| EF2388 | Hypothetical protein |  | 10 | (100) | 11 | (20) | 5E-05 |
| EF2389 | Hypothetical protein |  | 10 | (100) | 12 | (22) | 8E-05 |
| EF2662 | Choline binding protein |  | 7 | (70) | 2 | (4) | 1E-04 |
| EF2807 | Hypothetical protein | *p06* | 6 | (60) | 4 | (7) | 4E-03 |
| EF3099 | Transporter accessory protein, putative |  | 10 | (100) | 13 | (24) | 1E-04 |
| EF3101 | Conserved domain protein |  | 10 | (100) | 10 | (19) | 3E-05 |
| EF3102 | Hypothetical protein |  | 10 | (100) | 9 | (17) | 1E-05 |
| EF3103 | Membrane protein, putative |  | 10 | (100) | 9 | (17) | 1E-05 |
| EF3104 | ABC transporter, ATP-binding protein |  | 10 | (100) | 9 | (17) | 2E-05 |
| EF3105 | Hypothetical protein |  | 10 | (100) | 10 | (19) | 3E-05 |
| EF3153 | Conserved hypothetical protein |  | 8 | (80) | 9 | (17) | 2E-03 |
| EF3154 | Conserved hypothetical protein |  | 10 | (100) | 7 | (13) | 4E-06 |
| EF3155 | Conserved hypothetical protein |  | 8 | (80) | 1 | (2) | 3E-06 |
| EF3161 | Hypothetical protein |  | 10 | (100) | 18 | (33) | 9E-04 |
| EF3217 | Helicase, putative | New genomic islet | 10 | (100) | 3 | (6) | 3E-07 |
| EF3218 | Mutator MutT protein, putative | New genomic islet | 10 | (100) | 5 | (9) | 7E-07 |
| EF3220 | Hypothetical protein | New genomic islet | 8 | (80) | 3 | (6) | 3E-05 |
| EF3221 | Transcriptional regulator, Cro/CI family | New genomic islet | 8 | (80) | 2 | (4) | 1E-05 |
| EF3222 | Hypothetical protein | New genomic islet | 8 | (80) | 2 | (4) | 1E-05 |
| EF3223 | Hypothetical protein | New genomic islet | 9 | (90) | 3 | (6) | 2E-06 |
| EF3224 | Hypothetical protein | New genomic islet | 8 | (80) | 2 | (4) | 1E-05 |
| EF3225 | Conserved hypothetical protein | New genomic islet | 8 | (80) | 2 | (4) | 1E-05 |
| EF3226 | Rep protein | New genomic islet | 8 | (80) | 2 | (4) | 1E-05 |
| EF3227 | Conserved hypothetical protein | New genomic islet | 8 | (80) | 2 | (4) | 1E-05 |
| EF3241 | Abortive phage resistance protein, putative |  | 10 | (100) | 18 | (33) | 9E-04 |
| EF3242 | Abortive phage resistance protein, putative |  | 10 | (100) | 17 | (31) | 6E-04 |
| EF3243 | Hypothetical protein |  | 10 | (100) | 18 | (33) | 1E-03 |
| EF3248 | Hypothetical protein |  | 8 | (80) | 5 | (9) | 2E-04 |
| EF3250 | Hypothetical protein |  | 8 | (80) | 9 | (17) | 2E-03 |
| EF3251 | Hypothetical protein |  | 8 | (80) | 9 | (17) | 2E-03 |
| EF3252 | Hypothetical protein |  | 8 | (80) | 1 | (2) | 3E-06 |
| EFA0028 | Conserved domain protein | pTEF1 | 10 | (100) | 22 | (41) | 6E-03 |
| EFB0014 | Conserved hypothetical protein | pTEF2 | 10 | (100) | 19 | (35) | 1E-03 |
| EFB0015 | Hypothetical protein | pTEF2 | 9 | (90) | 16 | (30) | 4E-03 |
| EFB0016 | Hypothetical protein | pTEF2 | 9 | (90) | 17 | (31) | 6E-03 |
| EFB0019 | Conserved domain protein | pTEF2 | 9 | (90) | 17 | (31) | 6E-03 |
