# Supplementary material for: Comparative genomic analysis reveals significant enrichment of mobile genetic elements and genes encoding surface structure-proteins in hospital-associated clonal complex 2 Enterococcus faecalis
Source: BMC Microbiol. 2011 Jan 4;11:3. doi: 10.1186/1471-2180-11-3 (PMC3022643; doi:10.1186/1471-2180-11-3)
Supplement: Additional file 5 — Amino acid alignment of HMPREF0346_1863 in Enterococcus faecalis HH22 and its homologue in E. faecalis TX0104. An amino acid alignment of HMPREF0346_1863 in Enterococcus faecalis HH22 and its homologue in E. faecalis TX0104. [file 1471-2180-11-3-S5.DOC]

**Additional file 5. Amino acid alignment of HMPREF0346_1863 in *Enterococcus faecalis* HH22 and its homologue in *E. faecalis* TX0104.** The underlined region corresponds to a gap between ORFs HMPREF0348_0427 and HMPREF0348_0428 in the *E. faecalis* TX0104 draft genome, which was sequenced in the present study.

TX0104 1 MKKFLNLCIFYVIRVKNKIKYNFKEEEMIKKILFGVVCIFAFGGMAITAFADDTLPIYGS 60

MKKFLNLCIFYVIRVKNKIKYNFKEEEMIKKILFGVVCIFAFGGMAITAFADDTLPIYGS

HH22 1 MKKFLNLCIFYVIRVKNKIKYNFKEEEMIKKILFGVVCIFAFGGMAITAFADDTLPIYGS 60

TX0104 61 RIWFDLNGNGIQDQNEPSAPAIHFDKLAFTNKDLTVGFDYPGNNHLNAGSTTTPINSATA 120

RIWFDLNGNGIQDQNEPSAPAIHFDKLAFTNKDLTVGFDYPGNNHLNAGSTTTPINSATA

HH22 61 RIWFDLNGNGIQDQNEPSAPAIHFDKLAFTNKDLTVGFDYPGNNHLNAGSTTTPINSATA 120

TX0104 121 VIEPKSAWVKQNLNKDWSEITDKAMETDDWTEYESVSKQFSDANPLMDNAEVPYRTGFGN 180

VIEPKSAWVKQNLNKDWSEITDKAMETDDWTEYESVSKQFSDANPLMDNAEVPYRTGFGN

HH22 121 VIEPKSAWVKQNLNKDWSEITDKAMETDDWTEYESVSKQFSDANPLMDNAEVPYRTGFGN 180

TX0104 181 LNLANWIAQNVPEDSSYIN-----PSQLPKWLTITESNKASIVNSSQFSADGFYYFDNKA 235

LNLANWIAQNVPEDSSYIN PSQLPKWLTITESNKASIVNSSQFSADGFYYFDNKA

HH22 181 LNLANWIAQNVPEDSSYINYIYINPSQLPKWLTITESNKASIVNSSQFSADGFYYFDNKA 240

TX0104 236 PLQTVSGYYNLDGTFTESNDTQNPYVHSYAIANLGLIPHASIKLDMTTEQKISNPNKEIT 295

PLQTVSGYYNLDGTFTESNDTQNPYVHSYAIANLGLIPHASIKLDMTTEQKISNPNKEIT

HH22 241 PLQTVSGYYNLDGTFTESNDTQNPYVHSYAIANLGLIPHASIKLDMTTEQKISNPNKEIT 300

TX0104 296 VTYTVKNDGTSDLENITLSDIDFPAFNLKSGEEKTFSVAQIPNQQGIINTTVQGDLNYYY 355

VTYTVKNDGTSDLENITLSDIDFPAFNLKSGEEKTFSVAQIPNQQGIINTTVQGDLNYYY

HH22 301 VTYTVKNDGTSDLENITLSDIDFPAFNLKSGEEKTFSVAQIPNQQGIINTTVQGDLNYYY 360

TX0104 356 DQVYLNPDTGETSTTPQKPMHLLTVTDDKQVTVTYPTTKQSTITVRYMDEEGNQLIDPIT 415

DQVYLNPDTGETSTTPQKPMHLLTVTDDKQVTVTYPTTKQSTITVRYMDEEGNQLIDPIT

HH22 361 DQVYLNPDTGETSTTPQKPMHLLTVTDDKQVTVTYPTTKQSTITVRYMDEEGNQLIDPIT 420

TX0104 416 KTDIVGKEYSTEQKTFDGYQFEKLTGNASGVFTENDQEIVYVYKKIDVLKEENKAQINKT 475

KTDIVGKEYSTEQKTFDGYQFEKLTGNASGVFTE+DQEIVYVYKKIDVLKEENKAQINKT

HH22 421 KTDIVGKEYSTEQKTFDGYQFEKLTGNASGVFTESDQEIVYVYKKIDVLKEENKAQINKT 480

TX0104 476 SNTEFKEENKVNDSVKMDNTSTGKKIDKSLPRTGFNNNLVLNTLGSALLVVSFVGFTVVF 535

SNTEFKEENKVNDSVKMDNTSTGKKIDKSLPRTGFNNNLVLNTLGSALLVVSFVGFTVVF

HH22 481 SNTEFKEENKVNDSVKMDNTSTGKKIDKSLPRTGFNNNLVLNTLGSALLVVSFVGFTVVF 540

TX0104 536 VIKRLKQDK 544

VIKRLKQDK

HH22 541 VIKRLKQDK 549
